# Supplementary figures and images for: Two Waves of Specific B Cell Memory Immunoreconstruction Observed in Anti-HHV1–3 IgG Kinetics after Hematopoietic Stem Cell Transplantation
Source: Biomedicines. 2024 Mar 3;12(3):566. doi: 10.3390/biomedicines12030566 (PMC10968319; doi:10.3390/biomedicines12030566)

## Slide 1
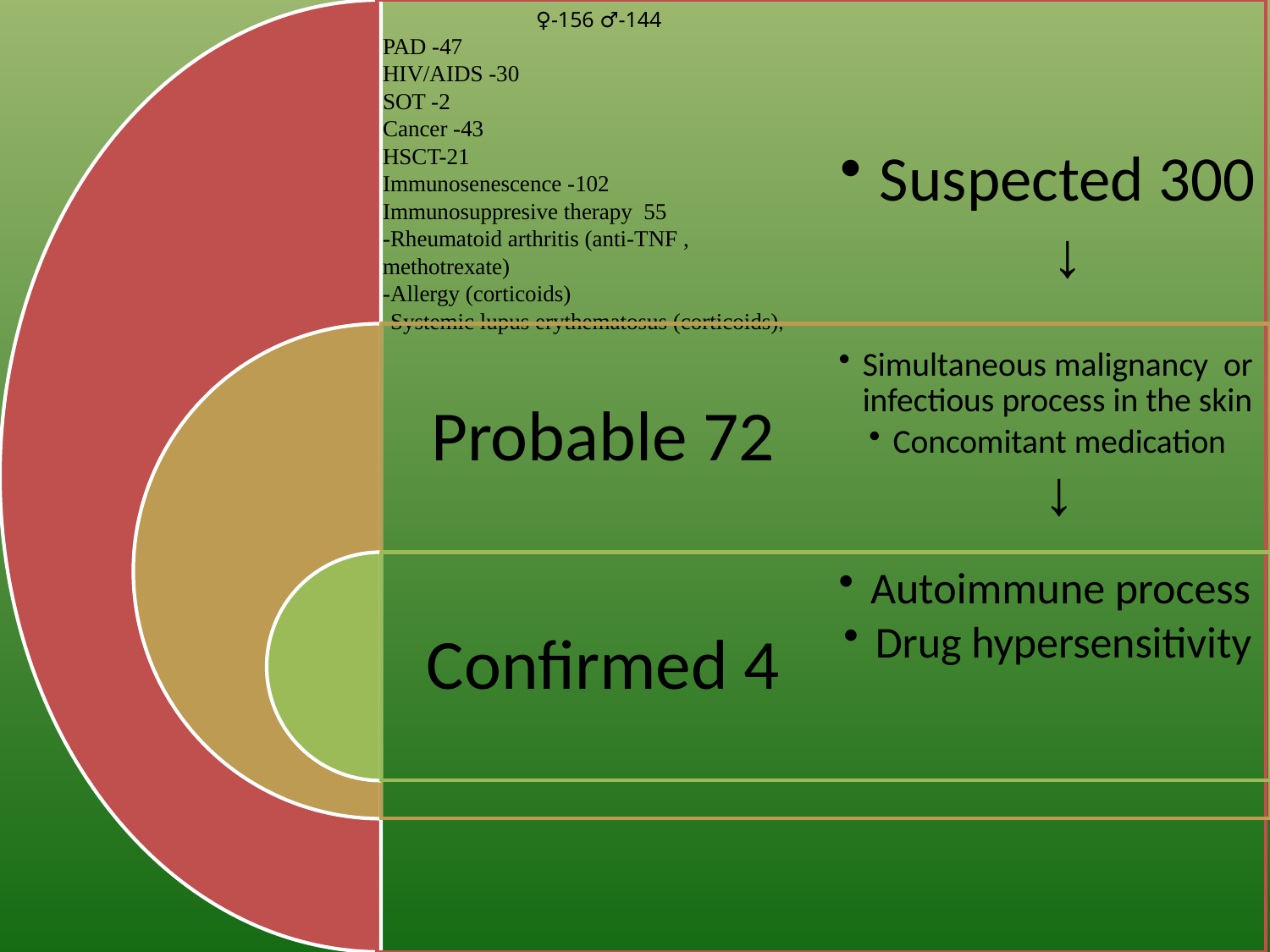

Supplement: Supplementary file 1 [file biomedicines-12-00566-s001.zip › SUPPLEMENTARY MATERIAL -PATIENTS SELECTION and diagnosis confirmation.pptx]
